# Outcome Comparison of Posterior Lumbar Fusion Versus Posterior Lumbar Interbody Fusion for Lumbar Degenerative Spondylolisthesis

**DOI:** 10.7759/cureus.91071

**Published:** 2025-08-26

**Authors:** Zia ur Rehman, Muhammad Aamir, Bilal Khan, Bashir Ullah, Muhammad Sohaib Khan

**Affiliations:** 1 Neurosurgery Department, Medical Teaching Institution (MTI) Lady Reading Hospital, Peshawar, PAK

**Keywords:** clinical outcomes, posterior lumbar fusion(plf), posterior lumbar interbody fusion(plif), radiological fusion, spinal fusion, spondylolisthesis

## Abstract

Background: Lumbar degenerative spondylolisthesis is a common spinal condition characterized by the anterior slippage of a vertebra over the one below due to degenerative changes. It often leads to chronic low back pain, radiculopathy, and spinal stenosis. Surgical intervention is typically considered when conservative treatments fail, with posterior lumbar fusion (PLF) and posterior lumbar interbody fusion (PLIF) being two widely used techniques.

Objective: To compare the clinical and radiological outcomes along with complication rates, among patients who underwent PLF and PLIF for lumbar degenerative spondylolisthesis.

Methodology: This prospective cohort study was conducted on 100 patients with lumbar spondylolisthesis who underwent either PLF (n=50) or PLIF (n=50). Preoperative clinical features, including low back pain, radicular symptoms, and neurologic deficits, were recorded. Postoperative outcomes at six months included low back pain, Oswestry Disability Index (ODI), radiological fusion (Bridwell grade), infection, hardware failure, and reoperation. Categorical data were compared using Chi-square tests and logistic regression was applied to determine independent associations.

Results: Postoperative low back pain was reported by 18 (36.0%) patients in the PLIF group compared to 42 (84.0%) patients in the PLF group (p < 0.001). PLIF patients showed superior functional outcomes with more achieving minimal disability on the ODI (80% vs. 52%, p = 0.007) and better radiological fusion (Grade 1 fusion in 60% vs. 24%, p < 0.001). Reoperation was significantly more frequent in the PLF group (24% vs. 4%, p = 0.004). Multivariate logistic regression confirmed PLIF as an independent predictor of improved outcomes.

Conclusion: PLIF demonstrated superior clinical and radiological outcomes with fewer reoperations compared to PLF at six-month follow-up. These findings support the preference for PLIF in selected cases of lumbar spondylolisthesis.

## Introduction

The term spondylolisthesis originates from the Greek word “olisthanein,” meaning “to slip,” and it refers to the anterior or posterior displacement of one vertebral body relative to another, resulting in segmental spinal instability [1,2]. Spondylolisthesis is broadly classified into six categories: isthmic, traumatic, degenerative, pathological, dysplastic, and postoperative, with degenerative spondylolisthesis (DS) being the most prevalent type, particularly in the elderly population [1,2].

The prevalence of DS exhibits notable age- and gender-related differences. It is relatively uncommon in individuals under 50 years of age, but its incidence increases significantly thereafter. Notably, women demonstrate a more rapid and higher prevalence of DS beyond the age of 50 compared to men, likely due to hormonal and biomechanical changes associated with aging [3].

Surgical intervention, particularly lumbar fusion, is frequently indicated in adults suffering from chronic, functionally limiting symptoms of degenerative disc disease (DDD) and lumbar spondylolisthesis that are refractory to conservative measures. Various lumbar fusion techniques are employed, including anterior lumbar interbody fusion (ALIF), posterior lumbar interbody fusion (PLIF), transforaminal lumbar interbody fusion (TLIF), and posterior lumbar fusion (PLF), each with its specific indications and biomechanical advantages [4,5].

PLF focuses on achieving spinal stability by inducing fusion between adjacent vertebrae through a posterior approach without entering the disc space. In contrast, PLIF involves direct access to the intervertebral disc space via a posterior incision, allowing the placement of interbody cages and bone grafts to facilitate disc height restoration and enhanced fusion rates [6]. Both techniques have demonstrated efficacy in improving pain and functional outcomes; however, PLIF has been associated with superior long-term results in certain patient populations due to its ability to restore spinal alignment and disc height more effectively [7].

Despite comparable operative time and intraoperative blood loss between PLF and PLIF, PLIF may offer additional advantages concerning sagittal balance correction and structural restoration of the spinal column, which are crucial for optimal long-term functional outcomes [8,9].

Degenerative spondylolisthesis commonly affects the elderly and often necessitates surgical intervention when conservative measures fail. PLF and PLIF are two widely used techniques, yet their comparative effectiveness remains debated. Evaluating both approaches can inform optimal surgical decision-making. This study aims to assess functional and radiological outcomes of PLF versus PLIF in managing lumbar degenerative spondylolisthesis.

## Materials and methods

This prospective cohort study was conducted in the Neurosurgery Department of the MTI Lady Reading Hospital, Peshawar, a tertiary care referral center for the province. Patient allocation to PLF or PLIF procedures was based on the operating surgeon’s discretion, taking into account patient anatomy, degree of vertebral instability, and clinical presentation. Ethical approval for the study was obtained from the hospital’s Institutional Review Board (Ref no: 326/LRH/MTI). Written informed consent was obtained from all participants prior to enrollment.

Patients aged 18 years or older undergoing elective posterior lumbar fusion for radiologically confirmed Grade I or II isthmic or degenerative spondylolisthesis at the L4-L5 or L5-S1 level were enrolled from October 2023 to January 2025. Inclusion criteria included chronic low back pain, radiculopathy, neurogenic claudication, or neurological deficits unresponsive to conservative management, with a minimum postoperative follow-up of six months. Exclusion criteria were prior lumbar instrumentation or multi-level fusion, high-grade spondylolisthesis (Grade III or higher), spinal infections, tumors, trauma, congenital spinal anomalies, severe osteoporosis (T-score ≤ -2.5), incomplete clinical or radiological data, or loss to follow-up before six months.

Patients were categorized into two groups based on the surgical technique employed: PLF or PLIF. All procedures were performed through an open posterior approach. For PLIF, standard titanium interbody cages were used in all cases. Autologous bone graft harvested from the iliac crest was applied in both PLF and PLIF groups. No allografts, bone morphogenetic proteins, or other osteobiologics were used. Supplemental instrumentation was applied where clinically indicated.

The sample size was calculated using a one-sided hypothesis test for two population proportions, with an anticipated effect size based on previous literature (p₁ = 0.93, p₂ = 0.68) [8]. At a 5% level of significance and 90% power, the minimum required sample size per group was 42. To enhance study power, accommodate potential data loss, and improve precision of estimated effect sizes, 50 patients were enrolled in each group (total n = 100).

Demographic and baseline clinical data were collected, including age, gender, smoking status, and presence of comorbidities (e.g., diabetes). Preoperative clinical features such as low back pain, radicular pain, neurogenic claudication, neurological deficits, and any history of previous spinal surgery were documented. Radiological evaluation included the type and grade of spondylolisthesis (isthmic vs. degenerative; Grade I or II).

Postoperative follow-up was conducted for a minimum of six months and a maximum of 18 months. Clinical outcome measures included the Visual Analog Scale (VAS) for pain and the Oswestry Disability Index (ODI) for functional status. Radiological fusion was assessed at the six-month follow-up using the Bridwell grading system. All patients underwent routine computed tomography (CT) scans in addition to standard anteroposterior and lateral radiographs to evaluate fusion status. Postoperative complications, including surgical site infection, hardware failure, and the need for reoperation, were recorded.

All data were entered and analyzed using IBM SPSS Statistics version 23 (IBM Corp, Armonk, NY, USA). Continuous variables such as age, VAS, and ODI scores were presented separately for the PLF and PLIF groups as mean ± standard deviation (SD) or median with interquartile range (IQR), depending on normality assessed by the Shapiro-Wilk test. Categorical variables, including gender, comorbidities, type and grade of spondylolisthesis, preoperative symptoms, radiological fusion, postoperative complications, and reoperation rates, were presented as frequencies and percentages for each group. Comparative analyses between the two groups were conducted using the chi-square test for categorical variables, and the independent t-test or Mann-Whitney U test for continuous variables, as appropriate. A p-value of ≤0.05 was considered statistically significant.

## Results

The study included 100 patients, evenly divided into two groups: 50 patients underwent PLF and 50 underwent PLIF. The average age of the patients was 51.4 ± 6.1 years. Other demographic and clinical characteristics such as gender distribution, smoking status, comorbidities, history of previous spine surgery, preoperative symptoms (low back pain, radicular pain, neurogenic claudication, neurologic deficit) and spondylolisthesis details (type, grade, level) compared between PLF and PLIF groups are summarized in Table 1 below.

**Table 1:** Preoperative Clinical and Radiological Characteristics Between PLF and PLIF Groups (n = 100) PLF: posterior lumbar fusion, PLIF: posterior lumbar interbody fusion

Variable	PLF Group (n = 50)	PLIF Group (n = 50)
Gender		
Male	29 (58.0%)	27 (54.0%)
Female	21 (42.0%)	23 (46.0%)
Smoker		
Yes	13 (26.0%)	17 (34.0%)
No	37 (74.0%)	33 (66.0%)
Comorbidities		
Yes	4 (8.0%)	1 (2.0%)
No	46 (92.0%)	49 (98.0%)
History of Previous Spine Surgery		
Yes	5 (10.0%)	7 (14.0%)
No	45 (90.0%)	43 (86.0%)
Low Back Pain Before Surgery		
Yes	31 (62.0%)	19 (38.0%)
No	19 (38.0%)	31 (62.0%)
Radicular Pain Before Surgery		
Yes	21 (42.0%)	22 (44.0%)
No	29 (58.0%)	28 (56.0%)
Neurogenic Claudication		
Yes	34 (68.0%)	31 (62.0%)
No	16 (32.0%)	19 (38.0%)
Neurologic Deficit Before Surgery		
Yes	4 (8.0%)	1 (2.0%)
No	46 (92.0%)	49 (98.0%)
Type of Spondylolisthesis		
Degenerative	36 (72.0%)	17 (34.0%)
Isthmic	14 (28.0%)	33 (66.0%)
Grade of Listhesis		
Grade I	22 (44.0%)	20 (40.0%)
Grade II	28 (56.0%)	30 (60.0%)
Level of Listhesis		
L4-L5	29 (58.0%)	22 (44.0%)
L5-S1	21 (42.0%)	28 (56.0%)

Postoperative low back pain was significantly more common in the PLF group (84%) compared to the PLIF group (36%) (p < 0.001). Functional outcomes, assessed using the ODI, revealed that a higher proportion of PLIF patients had minimal disability, whereas moderate to severe disability was more frequent in the PLF group (p = 0.007). Radiological fusion success at six months was higher in the PLIF group, with more patients achieving Bridwell grade 1 fusion compared to PLF. Conversely, graft lucency (grade 3 fusion) was more common in the PLF group. Reoperation rates were significantly higher in the PLF group (24% vs. 4%) (p = 0.004), while hardware failure rates were comparable between the two groups.

For detailed numerical results, refer to Table 2. Statistical comparisons were performed using the Chi-square test. A p value of < 0.05 was considered significant. 

**Table 2:** Comparison of Postoperative Outcomes and Complications Between PLF and PLIF Groups (n=100) PLF: posterior lumbar fusion, PLIF: posterior lumbar interbody fusion, ODI: Oswestry Disability Index

Variable	PLF (n=50)	PLIF (n=50)	p-value	Odds Ratio (95% CI)
Low Back Pain After Surgery			<0.001	0.107 (0.041 – 0.277)
Yes	42 (84%)	18 (36%)		PLF cohort: 0.375 (0.247 – 0.569)
No	8 (16%)	32 (64%)		PLIF cohort: 3.500 (1.843 – 6.648)
ODI Score Postoperative			0.007	Not computable (3 categories)
0–20% (Minimal Disability)	26 (52%)	40 (80%)		
21–40% (Moderate Disability)	14 (28%)	8 (16%)		
41–60% (Severe Disability)	10 (20%)	2 (4%)		
Radiological Fusion (Bridwell Grade)			<0.001	Not computable (3 categories)
Grade 1 (Fused with remodeling)	12 (24%)	30 (60%)		
Grade 2 (Graft intact, no lucency)	9 (18%)	11 (22%)		
Grade 3 (Graft with lucency)	29 (58%)	9 (18%)		
Infection			0.646	1.532 (0.245 – 9.587)
Yes	3 (6%)	2 (4%)		PLF: 1.213 (0.576 – 2.552)
No	47 (94%)	48 (96%)		PLIF: 0.792 (0.266 – 2.359)
Hardware Failure			0.240	2.667 (0.492 – 14.445)
Yes	5 (10%)	2 (4%)		PLF: 1.476 (0.883 – 2.467)
No	45 (90%)	48 (96%)		PLIF: 0.554 (0.169 – 1.815)
Reoperation Required During Follow-up			0.004	7.579 (1.599 – 35.933)
Yes	12 (24%)	2 (4%)		PLF: 1.940 (1.409 – 2.670)
No	38 (76%)	48 (96%)		PLIF: 0.256 (0.070 – 0.936)

Multivariate logistic regression analysis was performed without covariate adjustment because baseline characteristics were statistically comparable between groups. The results revealed that PLIF was independently associated with significantly lower odds of postoperative low back pain (OR = 0.107, p < 0.001), superior ODI scores (p = 0.002), and higher rates of radiological fusion (p < 0.001). Reoperation was also significantly less likely in the PLIF group (OR = 7.579, p = 0.011). No significant differences were found in infection or hardware failure rates between the groups. Detailed numerical results are summarized in Table 3 below.

**Table 3:** Multivariate Logistic Regressions of Postoperative Outcomes Among Surgery Type ODI: Oswestry Disability Index

Outcome Variable	B (β)	S.E.	p-value	Exp(B) [OR]	Nagelkerke R²
Postoperative Low Back Pain	-2.234	0.485	<0.001	0.107	0.302
ODI Score (Postoperative)	1.359	0.448	0.002	—	0.116
Radiological Fusion (Bridwell Grade)	1.688	0.404	<0.001	—	0.195
Infection	0.427	0.936	0.648	1.532	0.006
Hardware Failure	0.981	0.862	0.255	2.667	0.036
Reoperation Required During Follow-up	2.025	0.794	0.011	7.579	0.157

## Discussion

The present study sought to compare clinical and radiological outcomes, as well as complication profiles, between PLF and PLIF in patients undergoing surgery for lumbar spondylolisthesis. The findings suggest that PLIF is associated with significantly improved postoperative outcomes in terms of low back pain relief, functional status, radiological fusion, and lower reoperation rates, without a statistically significant increase in postoperative complications such as infection or hardware failure.

Patients who underwent PLIF experienced significantly lower rates of postoperative low back pain compared to those in the PLF group (OR = 0.107, p < 0.001). This finding aligns with the results reported by Inamdar et al., who found that PLIF was more effective in reducing back pain [9]. In contrast, Kang et al. and Lee et al. did not observe a significant difference in postoperative pain between the two techniques, suggesting that adequate posterior decompression alone may suffice in select patients [10,11]. Also, similar results are reported by Aygün et al. and Alijani et al., who reported that PLIF patients had better postoperative pain scores than PLF patients [12,13].

Functional recovery, as measured by the ODI, was also more favorable in the PLIF group, with a significantly greater proportion of patients achieving minimal disability scores (p = 0.002). The superior outcomes in the PLIF cohort are likely attributable to enhanced segmental alignment and interbody load sharing, which facilitate biomechanical stability and earlier functional recovery. These findings are supported by Faizan et al., Levin et al., Khasin et al., and Habib et al. [14-17]. However, Said et al. reported no clinically meaningful difference in ODI scores at six months, emphasizing the need for longer-term evaluations [18]. A study by Farrokhi et al. demonstrated opposite results, quoting that PLF had better ODI scores than PLIF [19].

Radiological assessment revealed a markedly higher fusion rate in the PLIF group, with 60% of patients achieving Bridwell Grade 1 fusion compared to 24% in the PLF group (p < 0.001). This finding is consistent with the results of Said et al., Farrokhi et al., Fallatah et al., Liu et al., Li et al., and Zhu et al., who reported better fusion rates with PLIF due to the large surface area and compressive forces at the interbody space, promoting osteogenesis [18-23].

The need for reoperation was significantly higher among PLF patients (24%) than PLIF patients (4%), with multivariate analysis confirming PLIF as a protective factor (OR = 7.579, p = 0.011). This is in agreement with the findings of Said et al., Durand et al., and Guppy et al., who attributed the higher reoperation rate in PLF due to pseudarthrosis and graft subsidence [18,24,25]. In contrast, Tang et al. found no significant difference in reoperation rates, suggesting that patient selection criteria and surgical expertise may influence outcomes more than the fusion technique itself [26].

No statistically significant difference was observed in infection (p = 0.646) or hardware failure rates (p = 0.240) between the two groups. These findings are corroborated by Said et al., who demonstrated comparable complication rates across both surgical techniques in a matched cohort [18]. Nonetheless, Elias et al. reported a marginally higher risk of implant-related complications in PLIF due to the use of interbody cages and increased operative time, highlighting the importance of meticulous technique and perioperative protocols [27].

There was some variability in the distribution of aetiological diagnosis between the two groups, with degenerative spondylolisthesis predominating in the PLF group and isthmic spondylolisthesis more frequent in the PLIF group. Although this difference may raise concerns regarding outcome comparability, prior literature on low-grade spondylolisthesis indicates that fusion rates do not significantly differ based on the underlying aetiology. Our results should therefore be interpreted in light of this evidence.

Limitations

The study had a relatively small sample size and was conducted at a single center, which may limit the generalizability of the findings. Follow-up duration was restricted to six months, preventing long-term assessment of fusion durability and functional outcomes. Potential selection bias and unmeasured confounders, such as surgeon experience and rehabilitation protocols, may have influenced the observed outcomes. Another limitation of this study is the use of the Bridwell grading system for evaluating posterolateral fusion. Although this allowed for uniform comparison between PLIF and PLF, it may not be the most specific system for posterolateral fusion. Alternative systems, such as that described by Lenke et al., could provide additional granularity.

## Conclusions

In conclusion, PLIF demonstrated superior clinical and radiological outcomes compared to PLF in patients with lumbar spondylolisthesis. It was associated with reduced postoperative pain, improved functional status, and higher fusion rates. These benefits were achieved without a significant increase in infection or hardware-related complications. However, due to limitations, further multicenter studies should be performed with a larger sample size and an extended follow-up period to better understand the clinical and radiological outcomes of these two techniques.

## References

[1] Bydon M, Alvi MA, Goyal A (2019). Degenerative lumbar spondylolisthesis: definition, natural history, conservative management, and surgical treatment. Neurosurg Clin N Am.

[2] Margetis K, Gillis CC (2025). Spondylolisthesis. StatPearls [Internet].

[3] Wang YX, Káplár Z, Deng M, Leung JC (2017). Lumbar degenerative spondylolisthesis epidemiology: a systematic review with a focus on gender-specific and age-specific prevalence. J Orthop Translat.

[4] Spiker WR, Goz V, Brodke DS (2019). Lumbar interbody fusions for degenerative spondylolisthesis: review of techniques, indications, and outcomes. Global Spine J.

[5] Rathbone J, Rackham M, Nielsen D, Lee SM, Hing W, Riar S, Scott-Young M (2023). A systematic review of anterior lumbar interbody fusion (ALIF) versus posterior lumbar interbody fusion (PLIF), transforaminal lumbar interbody fusion (TLIF), posterolateral lumbar fusion (PLF). Eur Spine J.

[6] Skoblar M, Hedman T, Rogers AJ, Jasper GP, Beall DP (2023). Instrumented posterior arthrodesis of the lumbar spine: prospective study evaluating fusion outcomes in patients receiving an interspinous fixation device for the treatment of degenerative spine diseases. J Pain Res.

[7] Dantas F, Dantas FL, Botelho RV (2022). Effect of interbody fusion compared with posterolateral fusion on lumbar degenerative spondylolisthesis: a systematic review and meta-analysis. Spine J.

[8] Dehoux E, Fourati E, Madi K, Reddy B, Segal P (2004). Posterolateral versus interbody fusion in isthmic spondylolisthesis: functional results in 52 cases with a minimum follow-up of 6 years. Acta Orthop Belg.

[9] Inamdar DN, Alagappan M, Shyam L, Devadoss S, Devadoss A (2006). Posterior lumbar interbody fusion versus intertransverse fusion in the treatment of lumbar spondylolisthesis. J Orthop Surg (Hong Kong).

[10] Kang YN, Ho YW, Chu W, Chou WS, Cheng SH (2022). Effects and safety of lumbar fusion techniques in lumbar spondylolisthesis: a network meta-analysis of randomized controlled trials. Global Spine J.

[11] Lee GW, Lee SM, Ahn MW, Kim HJ, Yeom JS (2014). Comparison of posterolateral lumbar fusion and posterior lumbar interbody fusion for patients younger than 60 years with isthmic spondylolisthesis. Spine (Phila Pa 1976).

[12] Aygün H, Cakar A, Hüseyinoğlu N, Hüseyinoğlu U, Celik R (2014). Clinical and radiological comparison of posterolateral fusion and posterior interbody fusion techniques for multilevel lumbar spinal stabilization in manual workers. Asian Spine J.

[13] Alijani B, Emamhadi M, Behzadnia H (2015). Posterior lumbar interbody fusion and posterolateral fusion: analogous procedures in decreasing the index of disability in patients with spondylolisthesis. Asian J Neurosurg.

[14] Faizan SA, Masaud M, Khan ZM, Imran T, Bashir A (2023). Comparative study on the effectiveness of posterolateral fusion vs. interbody fusion in isthmic/degenerative spondylolisthesis. Pak J Neurol Surg.

[15] Levin JM, Tanenbaum JE, Steinmetz MP, Mroz TE, Overley SC (2018). Posterolateral fusion (PLF) versus transforaminal lumbar interbody fusion (TLIF) for spondylolisthesis: a systematic review and meta-analysis. Spine J.

[16] Khasin M, Malham GM, Biddau DT (2022). PLF vs PLIF: moving towards a gold standard in posterior lumbar spinal fusion surgery. Epworth HealthCare Research Month 2022.

[17] Habib HA (2014). Posterolateral fusion versus posterior interbody fusion in adult lumbar isthmic spondylolisthesis. Menoufia Med J.

[18] Said E, Abdel-Wanis ME, Ameen M, Sayed AA, Mosallam KH, Ahmed AM, Tammam H (2022). Posterolateral fusion versus posterior lumbar interbody fusion: a systematic review and meta-analysis of randomized controlled trials. Global Spine J.

[19] Farrokhi MR, Yadollahikhales G, Gholami M, Mousavi SR, Mesbahi AR, Asadi-Pooya AA (2018). Clinical outcomes of posterolateral fusion versus posterior lumbar interbody fusion in patients with lumbar spinal stenosis and degenerative instability: a randomized trial. Pain Physician.

[20] Fallatah S (2013). Posterior inter-body fusion in lumbar spine surgery: a systematic review. J Taibah Univ Med Sci.

[21] Liu XY, Qiu GX, Weng XS, Yu B, Wang YP (2014). What is the optimum fusion technique for adult spondylolisthesis-PLIF or PLF or PLIF plus PLF? A meta-analysis from 17 comparative studies. Spine (Phila Pa 1976).

[22] Li Y, Wu Z, Guo D, You H, Fan X (2020). A comprehensive comparison of posterior lumbar interbody fusion versus posterolateral fusion for the treatment of isthmic and degenerative spondylolisthesis: a meta-analysis of prospective studies. Clin Neurol Neurosurg.

[23] Zhu Z, Xuan A, Xu C, Wang C, He Q, Tang L, Ruan D (2024). Comparison of clinical efficacy in the treatment of lumbar degenerative disease: posterior lumbar interbody fusion, posterior lumbar fusion, and hybrid surgery. Int J Spine Surg.

[24] Durand WM, Quan T, Parekh Y (2025). A comparative analysis of revision rates in surgical treatments for lumbar isthmic spondylolisthesis. Global Spine J.

[25] Guppy KH, Royse KE, Norheim EP, Harris JE, Brara HS (2021). PLF versus PLIF and the fate of L5-S1: analysis of operative nonunion rates among 3065 patients with lumbar fusions from a regional spine registry. Spine (Phila Pa 1976).

[26] Tang AR, Chanbour H, Steinle AM (2023). Which approach leads to more reoperations in single-level, open, posterior lumbar fusion: transforaminal lumbar interbody fusion or posterolateral fusion alone?. Int J Spine Surg.

[27] Elias WJ, Simmons NE, Kaptain GJ, Chadduck JB, Whitehill R (2000). Complications of posterior lumbar interbody fusion when using a titanium threaded cage device. J Neurosurg.

